# A multimethod approach for cross-cultural training in an internal medicine residency program

**DOI:** 10.3402/meo.v18i0.20352

**Published:** 2013-05-16

**Authors:** Lisa J. Staton, Carlos Estrada, Mukta Panda, David Ortiz, Donna Roddy

**Affiliations:** 1 Department of Internal Medicine, University of Tennessee College of Medicine-Chattanooga, Chattanooga, TN, USA; 2 Birmingham Veterans Affairs Medical Center, Birmingham, AL, USA; 3 Department of Internal Medicine, University of Alabama-Birmingham, Birmingham, AL, USA; 4 BlueCross BlueShield of Tennessee, Chattanooga, TN, USA

**Keywords:** cultural competence, residency education, community resources, web-based curriculum, racial disparities, internal medicine

## Abstract

**Background:**

Cultural competence training in residency is important to improve learners’ confidence in cross-cultural encounters. Recognition of cultural diversity and avoidance of cultural stereotypes are essential for health care providers.

**Methods:**

We developed a multimethod approach for cross-cultural training of Internal Medicine residents and evaluated participants’ preparedness for cultural encounters. The multimethod approach included (1) a conference series, (2) a webinar with a national expert, (3) small group sessions, (4) a multicultural social gathering, (5) a Grand Rounds presentation on cross-cultural training, and (6) an interactive, online case-based program.

**Results:**

The program had 35 participants, 28 of whom responded to the survey. Of those, 16 were white (62%), and residents comprised 71% of respondents (*n*=25). Following training, 89% of participants were more comfortable obtaining a social history. However, prior to the course only 27% were comfortable caring for patients who distrust the US system and 35% could identify religious beliefs and customs which impact care. Most (71%) believed that the training would help them give better care for patients from different cultures, and 63% felt more comfortable negotiating a treatment plan following the course.

**Conclusions:**

Multimethod training may improve learners’ confidence and comfort with cross-cultural encounters, as well as lay the foundation for ongoing learning. Follow-up is needed to assess whether residents’ perceived comfort will translate into improved patient outcomes.

## Introduction

Previous recommendations to teach about racial and ethnic disparities in health care highlighted the need for institutions to evaluate, teach, and adapt their curricula (1). National organizations recommend that health care professionals remain sensitive to cultural diversity, receive cultural competence training, and recognize that biases, stereotypes, and preconceived perceptions may contribute to disparities (2). Exposure to cultural competence training early in residency may underscore the importance of professionalism and promote interpersonal skills among physicians both in practice and in training. To date, cultural competence training has been shown to improve attitudes, knowledge, and skills of participants and improve patients’ ratings of care (3). Training may also be associated with fewer liability claims (4).

A variety of curricular approaches have been used to teach cultural competence. A previous study used a novel approach of nominal group techniques to demonstrate a cognitive map which defined four neighborhoods of concepts to be included in curriculum development: patient's background, provider/health care, cross-culture, and resources to manage cultural diversity (5). Another describes systematic methods to cover key areas of cultural competency using the mnemonic PRACTICE (prevalence, risk, attitude, communication, testing, investigation, consent, and empowerment (6).

Still, it is challenging for providers to become experts of all cultures in an increasingly ethnically diverse United States. Thus, the most effective methods to teach and practice cultural competence remain elusive (3). As the best delivery methods remain undefined, it is likely that multiple strategies and curricular approaches will be needed. We sought to design a cultural competence course based on educational theory which builds on self-directed learning and multimedia instruction. This instruction is based on the ‘dual-channel’ theory, a cognitive theory that there are two separate channels (auditory and visual) for processing information, wherein each ‘channel’ (visual, auditory) has capacity for learning (7). Hence, we used several training methods. The objective of this study is to describe our experience using multiple educational approaches to teach cross-cultural medicine and to assess participants’ perception in an Internal Medicine residency program.

## Methods

We conducted our study in a community-based, university-affiliated Internal Medicine residency program. We requested a waiver from the University of Tennessee College of Medicine, Chattanooga Institutional Review Board. This project was a descriptive, observational evaluation of cultural competence teaching concepts which began in August 2010. The educational program included multiple instructional approaches. Program objectives were to:

1. improve comfort with cross-cultural encounters;
2. improve the ability to have a dialogue about one's culture;
3. increase awareness of health care disparities and
4. collaborate with community stakeholders.

As part of routine educational activities, residents were required to review online case-based modules (which are available in the public domain) to generate a discussion regarding barriers to care, cross-cultural communication, and racial and ethnic disparities.

### Curriculum description

We developed a week-long cultural competence curriculum; Exhibit 1 outlines the week-long curriculum goals and objectives. Although, the program was duplicated 1 year later, here we report the results from 2010. The residency program dedicated existing conference time for 1 week to underscore the importance of cultural competence in residency education. All residents were encouraged to attend. However, residents who were off site, not on duty, or who had competing patient issues were excused. All residents and students who were enrolled in the medicine residency program were required to review five online modules to document learning in core competencies, including patient care, medical knowledge, professionalism, interpersonal and communication skills, and systems-based learning and practice-based learning. Developing the curriculum required 4–6 weeks of advanced planning in order to invite guest speakers and coordinate the timing of the webinar. The planning was coordinated with the Departmental Chair and the Graduate Medical Education in order to utilize existing conference times. In preparation for the program, flyers and announcements were sent out via email and posted throughout the hospital.

**Exhibit 1:** Description of multimethod learning activities

Teaching method	Learning activity	Goals and objectives
Noon conference	Overview of cultural competence, health literacy, and health care disparities	Discuss goals and objectives for the week
		Highlight resources to teach models of care for culturally discordant encounters
		Define cultural competence concepts and definition
		Introduce skills to obtain a history
		Identify cross-cultural issues
		Discuss the role of health care literacy
Graduate medical education combined conference	Acculturation and enculturation	Discuss the importance of diversity in health care
		Encourage dialog among trainees
		Describe the values of respecting other cultures
		Define the process of adoption of surrounding culture
Webinar national expert and panel discussion	Review the goals of quality interactions	Augment access to education regarding cultural competence and identify point of care resources
		Identify tools to conduct a culturally competent history and medical examination
		Discuss how to work effectively with interpreter services
		Identify the impact of cultural issues on medical decision-making
		Resource: www.qualityinteractions.org/prod…/clinical_program_features.html
Grand rounds	National expert	Discuss the key concepts for developing a cultural competence curriculum
		Discuss how to organize critical domains used to guide development of a cultural competence curriculum
		Resource: Cultural Competency Online for Medical Practice (http://www.c-comp.org).
Panel discussion	Faculty administrators community employees diversity officer	Discuss role for workplace diversity
		Promote dialog among residents, faculty, and community partners
		Identify opportunities for collaboration
		Engage leadership in discussion
Small group discussions	Dialogue and desserts from around the world- multicultural luncheon	Discuss cultural background of participants
		Understand and share cultural norms
Self-study	Completion of online modules	Quality interactions

Multimethod learning activities included noon conferences, a webinar with a national expert, small group discussions, and a Grand Rounds discussing the development of Cultural Competency Online for Medical Practice (http://www.c-comp.org). Residents were expected to complete an interactive, online case-based training, Quality Interactions^®^ (http://www.qualityinteractions.org/). There were five online interactive modules which incorporated live clinical encounters to demonstrate different aspects of cross-cultural communication. A panel discussion and the multicultural social activity served as springboards for discussions.

### Curriculum assessment

We developed a 28-item evaluation tool based on domains from the Cross-Cultural Preparedness Survey, an 18-item survey with three components: general cross-cultural preparedness, general cross-cultural skillfulness, and cross-cultural language preparedness and skillfulness (8). The survey was administered online and was applicable for all the sessions (Survey Monkey. com LLC, Palo Alto, California, USA). The survey included demographic characteristics such as year of training, race, and gender. Each teaching component was evaluated on a five-point Likert scale to reflect agreement with the participants’ perception of the usefulness to improve, knowledge, attitudes, or skills. The target audience was internal medicine residents and students enrolled at the time of the program. Participants included medical students, residents, hospital staff, and academic and hospital leaders. There was variable participation in the sessions and not all participants completed every session since clinical and other duties sometimes precluded conference attendance. The survey was sent out on the last day of the course and participants were asked to complete within 1 week. There were no specific recruitment efforts for the study and no exclusion criteria. We used standard statistics (frequencies, percentages) to describe the main results.

## Results

Of 35 program participants considered as the target audience, 28 responded; data on non-responders were not available. Of those, 8 were female (31%) and 18 male (69%); 16 were white (62%), 5 African American (19%), and 5 Asian (19%). Twenty residents comprised 71% of respondents, two faculty (7%), two medical students (7%), and four others (15%: clinical staff and stakeholders such as hospital and university administrators, and industry employees). Most participants were first year (*n*=9, 32%) or second year (*n*=6, 21%) residents. The respondents’ participation is noted in Table 1. The highest rated session was the *Cultural Competency Overview, Health Literacy and Disparities in Women Health*; 39% of respondents (*n*=11) rated the session as strongly agree and 36% agreed (*n*=10) to the statement ‘improved my attitude regarding cultural competence and health disparities.’ Of the 28 respondents, many felt that the course improved their confidence in cross-cultural encounters as 33% (*n*=9) reported that they strongly agreed and 48% (*n*=13) agreed.

**Table 1:** Attendance and ratings by teaching activity

Learning activity	Respondents	Improved my knowledge or skill response agree, *n* (%)	Improved my knowledge or skill response strongly agree, *n* (%)
Overview of cultural competence, health literacy, and health care disparities	22 (79)	10 (36)	11 (39)
Acculturation and enculturation	21 (75)	15 (60)	5 (20)
Review the goals of quality interactions	33 (79)	14 (50)	7 (25)
National expert	19 (68)	9 (33)	10 (37)
Faculty administrators community employees diversity officer	23 (82)	11 (42)	8 (31)
Dialog and desserts from around the world – multicultural luncheon	13 (46)	NA	NA

Evaluations of domains were categorized as cultural knowledge, cultural attitudes, or cultural skills – all shown in Table 2. Across all questions, most found the activities to be helpful (>85% for all; see Table 2).

**Table 2:** Responses following program (%) (*n*=28)

Domain/item	Helpful, *n* (%)	Not helpful, *n* (%)	Not applicable, *n* (%)
Cultural knowledge – activity prepared me to:			
Feel comfortable identifying when a patient mistrusts the health care system or physicians	26 (92)	1 (4)	1 (4)
Feel prepared to care for new immigrants	26 (92)	1 (4)	1 (4)
Feel comfortable identifying cultural customs that might affect care	26 (92)	1 (4)	1 (4)
Feel comfortable identifying how patients make decisions with family	25 (89)	2 (7)	1 (4)
Cultural attitudes – activity prepared me to care for patients with:			
Cultural differences	26 (93)	0 (0)	2 (7)
Racial differences	26 (92)	1 (4)	1 (4)
Religious differences	25 (89)	2 (7)	1 (4)
Beliefs at odds with Western beliefs	26 (93)	0 (0)	2 (7)
Limited English	26 (92)	1 (4)	1 (4)
Cultural skills – activity prepared me to care for patients and:			
Determine how to address people from different culture	26 (93)	2 (7)	0 (0)
Take a social history	24 (89)	3 (11)	0 (0)
Assess the patient's understanding of the cause of illness	26 (93)	2 (7)	0 ()
Negotiate a treatment plan	24 (85)	1 (4)	3 (11)
Identify whether a patient can read and write English	25 (88)	1 (4)	2 (8)
Identify cultural customs which affect care	26 (92)	1 (4)	1 (4)

## Discussion

Internal Medicine residency program participants in a cultural competence education training course felt that the structured course helped prepare them for culturally competent care. Our study supports previous literature suggesting that integration of cultural competence concepts into other clinical teaching may be effective in reinforcing that this is not an ‘add-on’ topic for study (9). Training can lay the foundation for ongoing learning and has the potential to increase residents’ comfort with cross-cultural encounters in the clinical setting.

We opted to use multiple teaching strategies. Multimedia design principles have been shown to improve short-term retention among medical students, though further studies are needed to determine how these principles affect transfer of learning (10). Our results support previous work that is based on the social-cognitive perspective of self-directed learning, wherein curricular elements (patients’ cultural background, provider/health care skills, bias, cross-cultural communication, and resources to manage cultural diversity) parallel the theoretical framework. Learning any new skill requires four cognitive processes:

1. attention;
2. retention;
3. production; and
4. motivation.

In one study, learners were exposed to earlier versions of an oral presentation, which developed over time into more polished versions. Retention is increased by using ‘multichannel’ instruction, such as videos and open discussions (7). Not only were multiple teaching strategies used but also multiple stakeholders were involved. Involving community members may help improve learners’ confidence in their preparedness for cultural encounters (9). This approach, like other systems approaches, is unlikely to eliminate disparities. However, a systems approach to training interventions can improve disparities by changing attitudes and behaviors in leaders, caregivers, and health care organizations (11). Our study is limited by our small sample size and participant drop off in some sessions. Although residents demonstrated immediate increased confidence, future surveys are needed to determine long-term impact. For example, in the business world there is a widely used approach, the Kirkpatrick Model, which can be used to assess learners at different levels. Application of this model could help serve as a catalyst for additional applications. We could not only evaluate reaction and learning but we could also assess behaviors and outcome (12).

Nonetheless, our program design allowed us to employ multiple educational strategies to provide education and exposure. In previous studies, very few opportunities existed for cultural competence education and mentoring, highlighting the need for more training for residents (13). This project, as part of a regular educational session in an Internal Medicine residency training program was enthusiastically received by participants. Similar programs could be a first step in convening residents, staff, faculty administration, and community to facilitate discussion regarding cultural competence and health care disparities. Our multimethod approach could easily be implemented in other residency programs. Buy-in from stakeholders can be obtained given accreditation mandates and the challenges of changing demographics in the United States.

## Practice points

1. Recognize the need for cultural competence training in residency.
2. Improve learners’ confidence in their preparedness in cross-cultural encounters.
3. Discuss multimethod educational strategies to develop the curriculum to teach cultural competence.
4. Discuss implementation of a cultural competence curriculum in an Internal Medicine residency program.
5. Highlight results of the evaluation of the cultural competence curriculum.
